# A Rare TPM3-NTRK1 fusion in a fetal pelvic mass

**DOI:** 10.1016/j.radcr.2025.02.079

**Published:** 2025-03-15

**Authors:** Maryam Kazelka, Lei Shao, Marty McGraw, Jennifer Neville Kucera

**Affiliations:** a University of South Florida, Morsani College of Medicine, 560 Channelside Dr, Tampa, FL 33602, USA; b Nemours Children's Hospital, Department of Pathology, 6535 Nemours Pkwy, Orlando, FL 32827, USA; c Nemours Children's Hospital, Department of Radiology, 6535 Nemours Pkwy, Orlando, FL 32827, USA; d University of Central Florida, Department of Radiology, 6850 Lake Nona Blvd, Orlando, FL 32827, USA; e University of South Florida, Department of Radiology, 2 Tampa General Circle, STC 6102, Tampa, FL 33606, USA

**Keywords:** Congenital tumor, Spindle cell sarcoma, NTRK-rearrangement, TPM3-NTRK1 fusion

## Abstract

Early recognition and characterization of soft tissue tumors is important for proper fetal and maternal care. Here, we present sonographic, fetal and postnatal MRI, and pathological findings of a rare case of congenital NTRK-rearranged malignant spindle cell sarcoma with TPM3-NTRK1 fusion in a male fetus.

## Introduction

NTRK-rearranged spindle cell sarcomas are a rare and aggressive type of soft-tissue sarcoma with an infiltrative growth pattern. This tumor primarily affects children and young adults, favoring the extremities [1]. This rapidly growing cancer can damage organs during development due to mass effect and direct invasion in a growing fetus and potentially cause dangerous hemorrhage during birth.

## Case report

**Fig. 1:**
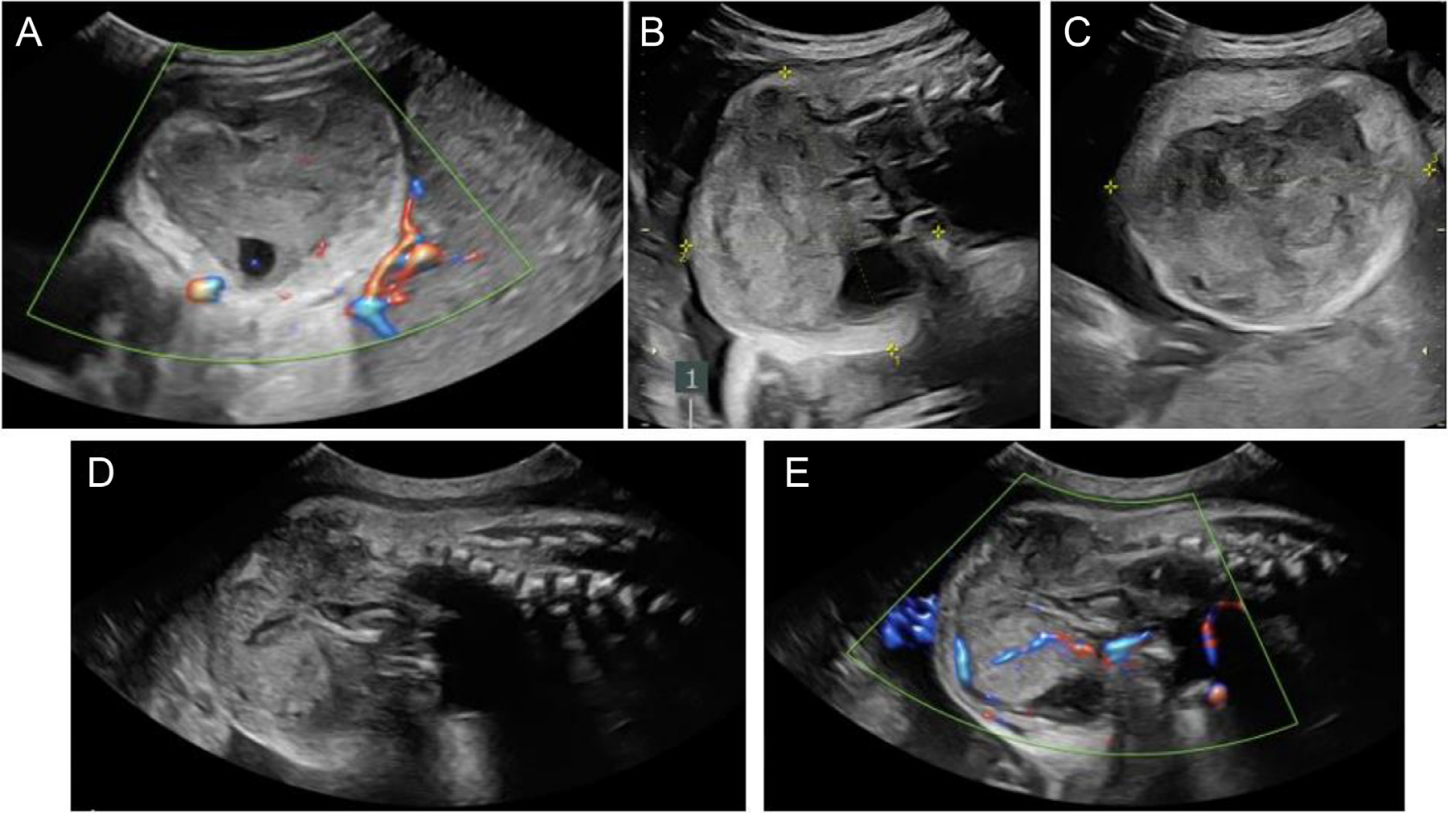
Fetal ultrasound performed at 34 weeks 6 days gestation. The transverse (A-C) and sagittal (D, E) planes demonstrate a mass exophytic to the distal spine. The mass can be seen extending from the distal aspect of the spine measuring 7.63 × 6.47 × 8.17 cm (B, C). Two-dimensional color mapping (A, E) demonstrates the vascular nature of the mass.

**Fig. 2:**
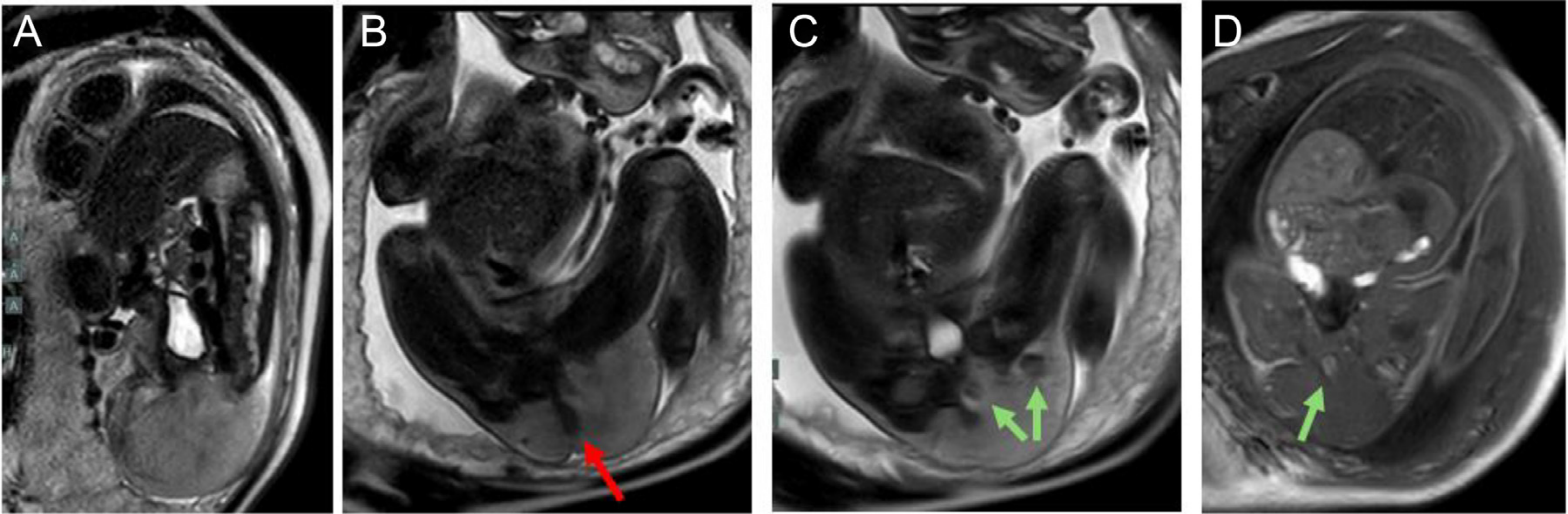
Fetal MRI performed at 35 weeks and 4 days gestation. Sagittal T2 weighted image (A) reveals a large mass centered on the pelvis but no extension into the spinal canal. Coronal T2 weighted images (B, C) show the displacement of the rectum (red arrow) with areas of hemorrhage (green arrows). The mass is hyperintense to skeletal muscle. Coronal T1 image (D) shows that the mass is isointense to skeletal muscle. Foci of hemorrhage are again noted (green arrow).

A 25-year-old healthy G2P1001 female presented at 34 weeks gestational age with growth restriction of a male fetus. The pregnancy was previously uncomplicated. There was no family history of congenital malformations, birth defects, chromosomal abnormalities, or learning disabilities. Use of tobacco, alcohol, and illicit drugs during the pregnancy was denied. Maternal labs were normal throughout pregnancy. NIPS genetic screening was declined. At the mother's 34 week prenatal visit, the abdominal circumference was measured and corresponded to the 1.9% percentile, consistent with fetal growth restriction. This finding prompted a detailed fetal evaluation. Prenatal ultrasound (Fig. 1) showed an exophytic, complex, vascular mass abutting the sacrum with a presumed diagnosis of sacrococcygeal teratoma (SCT) given its location. For further characterization, a fetal MRI was performed at 35 weeks gestation, which showed a heterogeneously enhancing, solid, exophytic pelvic/perineal mass abutting the sacrum (Fig. 2). Many features of the tumor were atypical for a sacrococcygeal teratoma. The mass was more homogenous than expected for a SCT, lacked cystic components that would normally been seen in a SCT, and involved the thigh but without definite involvement of the spine. It was also noted that the rectum was displaced posteriorly by the mass. At that time, alternative differential diagnoses were suggested: fibrosarcoma, rhabdomyosarcoma, myofibroma, and less likely atypical sacrococcygeal teratoma.

**Fig. 3:**
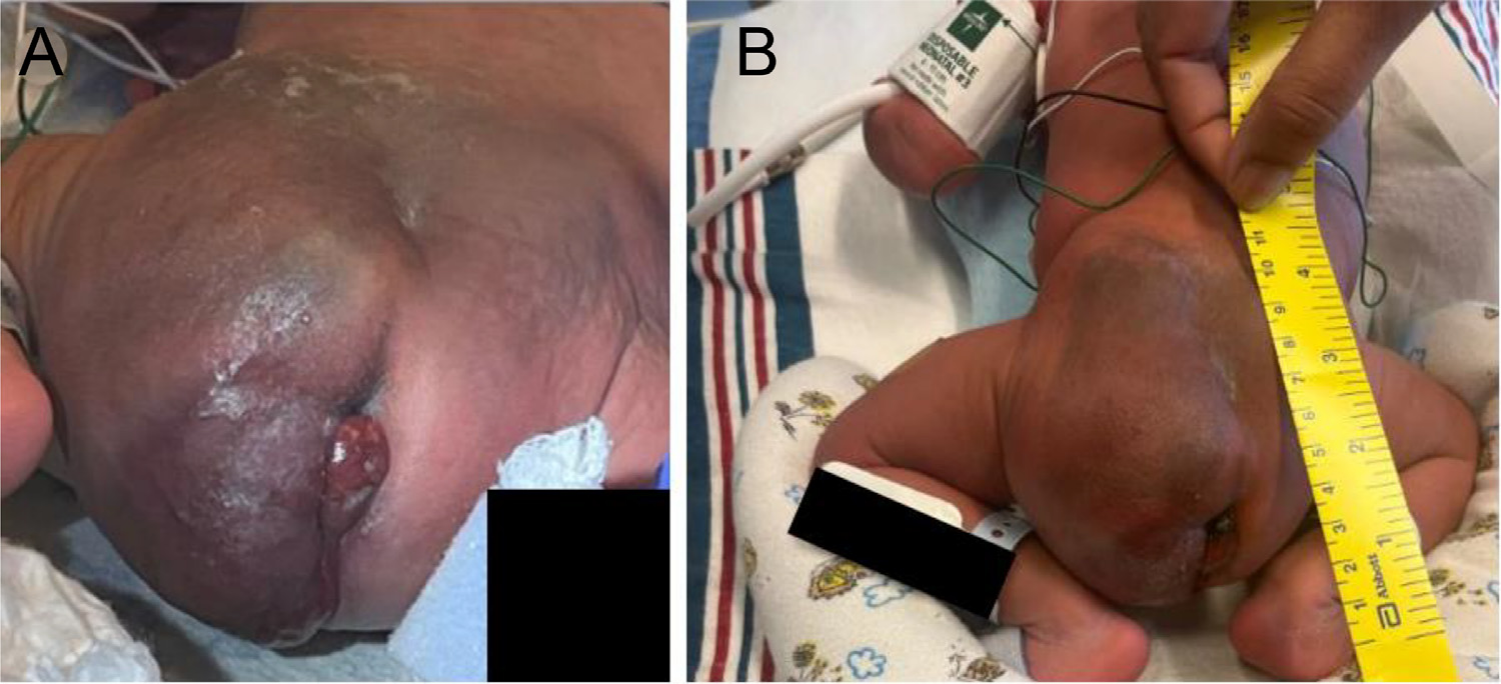
Infant presentation at birth showing a left pelvic and gluteal region mass (A, B). The overlying skin was friable but there was no active bleeding.

**Fig. 4:**
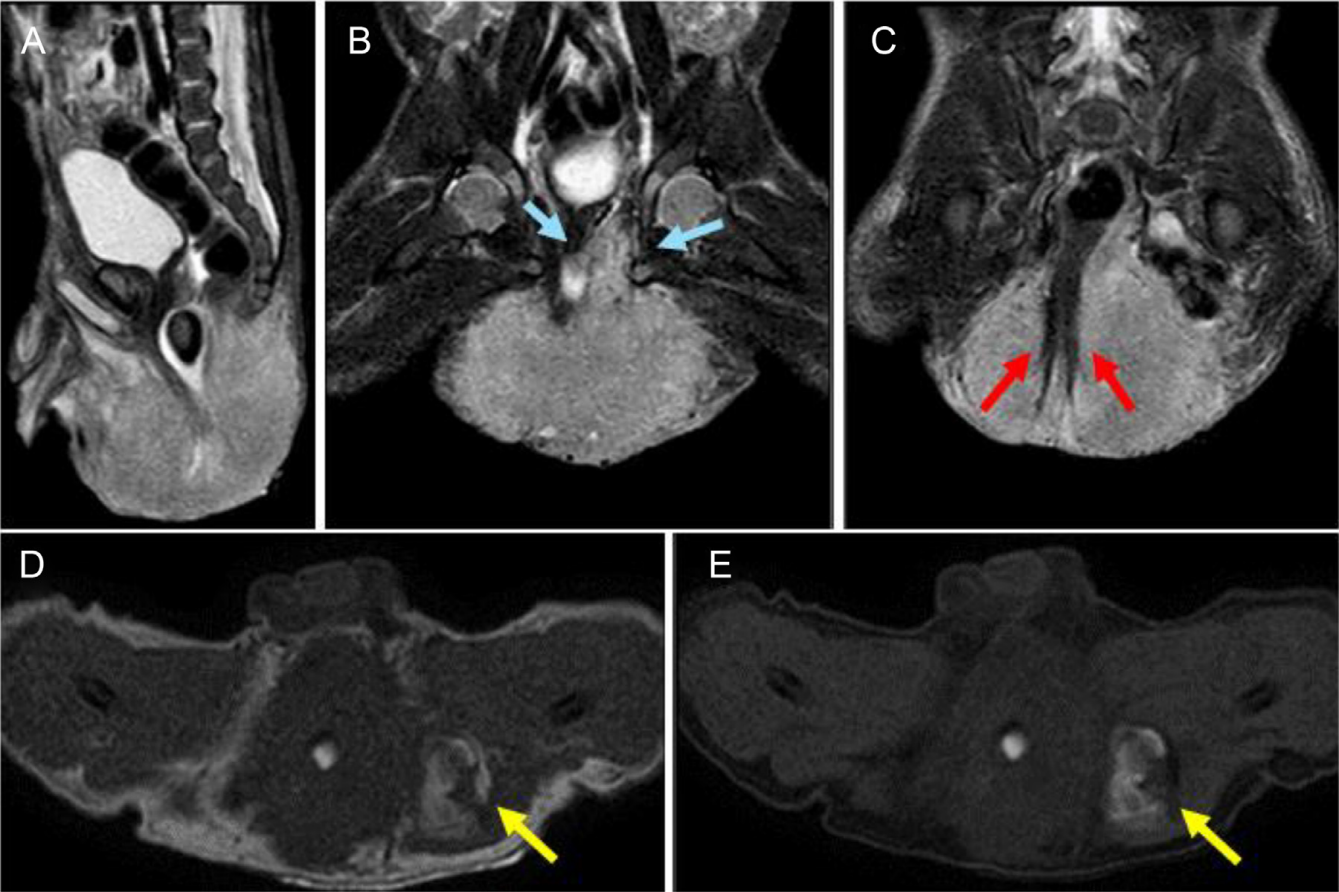
Postnatal MRI performed at 3 days old. Sagittal STIR (A) shows the pelvic mass exophytic to but not invading the spine. Coronal STIR imaging (B, C) shows tumor extension through the left inguinal canal (blue arrows). The mass displaces the rectum and anus which has a tethered appearance (red arrow). Axial T1 weighted image without (D) and with (E) fat saturation show hyperintense signal (yellow arrow) within the mass, which does not lose signal with fat saturation (yellow arrow), compatible with hemorrhage.

The baby was delivered via C-section at 37 weeks and 1 day gestation due to fetal distress. The large left pelvic mass was evident (Fig. 3). A postnatal MRI showed a large, exophytic, heterogeneously enhancing perineal/pelvic mass with infiltrative pattern that encircled the rectum with extension into the left gluteal and inguinal regions (Fig. 4). The mass was isointense to skeletal muscle on T1WI, hyperintense on fluid sensitive sequences, and demonstrated heterogeneous enhancement. The tumor demonstrated mass effect on multiple structures: deviating and tethering of the rectum to the right and stretching it inferiorly; displacing the anus posteriorly; uplifting the bladder; and abutting the base of the penis with no clear fat planes. Lack of spine involvement was confirmed.

**Fig. 5:**
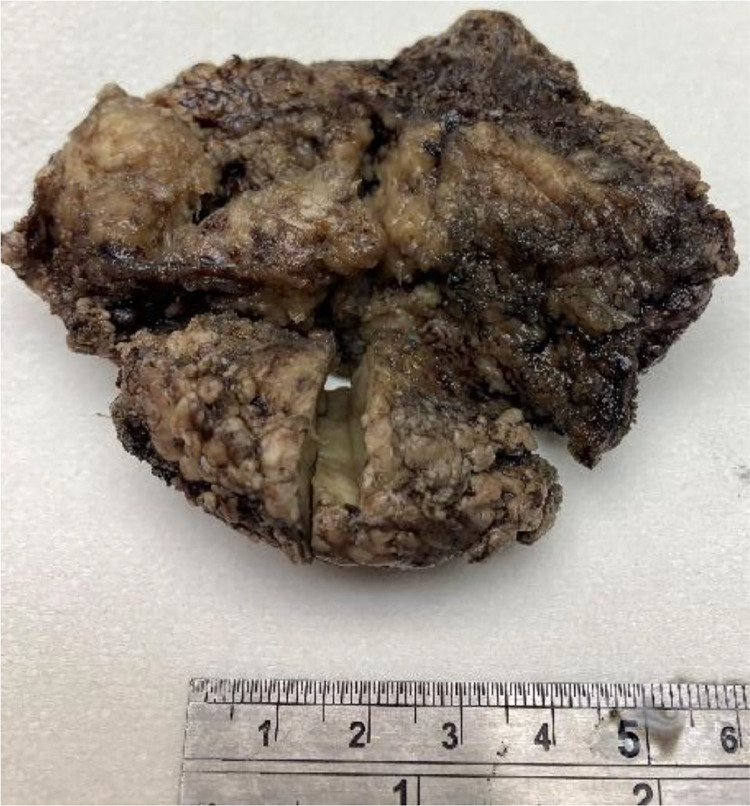
Formalin fixation of the specimen with ruler.

**Fig. 6:**
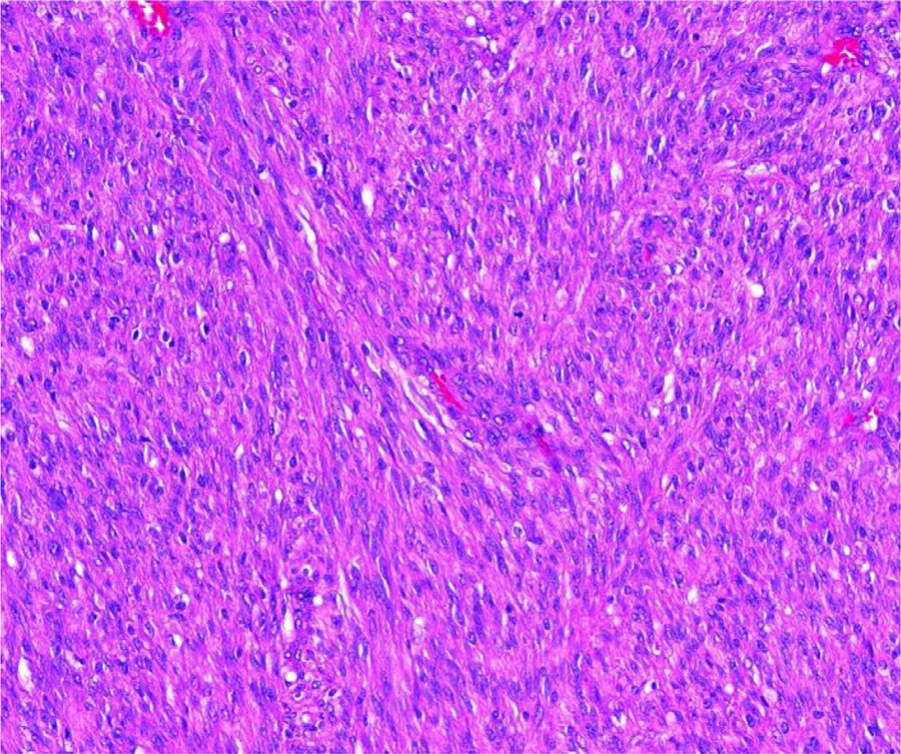
Histological examination reveals elongated, spindle shaped cells arranged in a fascicular pattern.

Surgical resection was performed when the baby was 6 days old. The specimen was fixed in formalin (Fig. 5). Pathology evaluation revealed a sarcoma that was diffusely infiltrative with NTRK reactivity by immunohistochemistry. Molecular studies by next-generation sequencing reported TPM3-NTRK1 fusion in the tumor. The tumor lacked the typical ETV6-NTRK3 fusion seen with >85% of infantile fibrosarcomas and focally tested positive for MyoD1 and Myogenin. The final pathology was congenital NTRK-rearranged malignant spindle cell sarcoma (Fig. 6). Following surgical resection, chemotherapeutic treatment with Larotrectinib, a first generation TRK inhibitor, was initiated.

## Discussion

Pediatric soft tissue sarcomas belong to a diverse group of tumors that arise from embryonic mesodermal tissues during their differentiation into various mesenchymal components of the body. These malignancies account for approximately 6%-8% of all cancers diagnosed in children under the age of 15 [2,3].

MRI is the modality of choice for characterization of soft tissue tumors due to its high contrast resolution. Generally, soft tissue sarcomas are large, complex, heterogeneously enhancing masses with irregular borders, an infiltrative growth pattern, and restricted diffusion. Spindle cell sarcomas usually appear isointense to skeletal muscle on TI weighted images and hyperintense on T2 weighted images. MRI is also better able to demonstrate mass effect and infiltration of surrounding tissue, which is important for guiding surgical resection planning. Edema may also be evident due to the mass effect, which is visualized well on T2 weighted images [4]. This spindle cell sarcoma with TPM3-NTRK1 fusion appeared as a homogenous mass with signal characteristics most similar to skeletal muscle with focal areas of hemorrhagic contents. The mass was revealed to be vascular on doppler ultrasound. The mass was hyperintense to skeletal muscle on T2 weighted images and isointense to skeletal muscle on T1 weighted images. Several other tumors can present similarly but may have distinguishing features on MRI that aid in diagnosis. Teratomas present as complex mixed cystic and solid masses typically with macroscopic fat and calcifications. Rhabdomyosarcomas and fibrosarcomas are both solid masses with an imaging appearance similar to spindle cell sarcomas and typically require biopsy for definitive diagnosis [4].

Although MRI is an incredible tool for visualizing and characterizing soft tissue tumors, an exact diagnosis may not be possible through imaging alone. Pathological examination after surgical resection or biopsy is extremely valuable in identifying these tumors. Spindle cell sarcomas are characterized by elongated spindle-shaped cells when observed under a microscope, resembling fibroblasts or smooth muscle cells arranged in fascicles or whorls. Immunohistochemical workup generally demonstrates reactivity to vimentin, desmin, myogenin, and MyoD1 markers. Our tumor stained positive for pan-TRK and CD30. Initially thought to be an infantile fibrosarcoma (IFS), cytogenetic FISH testing for confirmation of the NTRK fusion was found to be negative for the ETV6-NTRK3 fusion classically found in an IFS [5]. Further evaluation revealed a TPM3-NTRK1 fusion. Our tumor was ultimately found to be a very rare NTRK-rearranged spindle cell sarcoma with TPM3-NTRK1 fusion by next generation sequencing (NGS). NTRK fusions have been found to be more common in the spindle cell subtype of sarcomas than other subtypes with many fusion pairings having been recorded in the literature [[6], [7], [8]], but an NTRK-rearranged spindle cell sarcoma with a TPM3-NTRK is incredibly rare.

The NTRK (neurotrophic receptor tyrosine kinase) fused sarcomas are a rare subset of soft tissue sarcoma that can arise in various tissues, including bones, muscles, and connective tissues. NTRK gene fusions cause constant activation of transcription of TRK fusion proteins, which promote cell growth and survival. Although most cases arise sporadically, they may also be associated with predisposing syndromes including neurofibromatosis type 1 and Li-Fraumeni syndrome [9]. The TPM3-NTRK fusion has been reported in a case of a child with Bloom syndrome, with the fusion appearing in both BLM alleles [10]. In our case, the tumor had a spontaneous origin.

As these rapidly growing tumors are usually painless, symptoms produced by the tumors are more often due to the location and infringement on surrounding structures. Most commonly presenting in the genitourinary region in children, symptoms may include but are not limited to rectal displacement and compression leading to constipation or fecal incontinence; displacement and compression of the bladder leading to incontinence; penile compression leading to erectile dysfunction and infertility. In addition, these tumors may be painful.

Compared with other subtypes or sarcomas, NTRK rearranged spindle cell sarcomas in children are associated with a favorable outcome [1,11]. Treatment generally includes surgical resection and TRK inhibitors, such as Larotrectinib. The NTRK fusion makes cancer cells that contain this mutation an easy, specific target for chemotherapy. First-generation TRK inhibitors are generally well tolerated by patients due to a low toxicity profile [12,13]. While these inhibitors often provide effective disease control, NTRK fusion-positive cancers can develop resistance over time, often due to acquired mutations in the NTRK kinase domain. Some of the mutations that confer resistance can be combated with second-generation TRK inhibitors, such as LOXO-195 and TPX-0005 [14]. Radiation therapy is generally reserved for locally advanced or metastatic disease, which was not present in our patient.

## Conclusion

The radiologist should understand imaging features that may help distinguish more rare soft tissue tumors from sacrococcygeal teratomas. These tumors should be kept on the differential in any soft-tissue tumor in an infant, especially with areas showing hemorrhage on imaging. It is important for the pediatric radiologist to be familiar with fetal soft tissue tumors to help facilitate family counseling and delivery planning. Early recognition and accurate imaging are essential for timely diagnosis and optimizing treatment outcomes. Although an exact diagnosis may not be possible prenatally, a multidisciplinary approach ensures the best possible patient outcomes.

## Patient consent

Written informed consent for publication of their case was obtained from the patient.
